# Engaging a Community Advisory Board to Integrate Family Caregivers in Hospital-to-SNF Transitions

**DOI:** 10.1093/geroni/igaf122.317

**Published:** 2025-12-31

**Authors:** Alycia Bristol, Shinduk Lee, Brenda Luther, Gail Towsley

**Affiliations:** University of Utah, Salt Lake City, Utah, United States; University of Utah, Salt Lake City, Utah, United States; University of Utah, Salt Lake City, Utah, United States; University of Utah, Salt Lake City, Utah, United States

## Abstract

Transitions from hospitals to skilled nursing facilities (SNFs) are often complex and require careful planning with older adults and their family caregivers (FCGs; e.g., unpaid family or friends). Despite the numerous existing care transition interventions, few consider FCG involvement, contributing to poor communication, poor patient outcomes, increased readmission risks, and reduced care quality. Using the Integrated Research-Practice Partnerships Process Model, we formed a community advisory board (CAB) with members from hospitals (n = 2), SNF/post-acute (n = 2), and FCGs (n = 2). In three one-hour meetings, the CAB provided input on FCG integration and intervention protocols and materials to co-develop a patient- and family-centered intervention, Better Connections. A post-meeting survey gathered feedback on satisfaction and CAB structure. The CAB identified three phases for FCG involvement during hospital-SNF transitions: 1) predischarge hospital meeting; 2) planning for transfer; and 3) SNF initial care plan meeting. Key elements included improving communication, establishing points of contact, and identifying and fulfilling unmet education/transition needs. The CAB recommended developing conversation guides with questions, resources, and communication tips for FCGs and hospital/ SNF teams. Five of six surveys were completed. CAB members agreed or strongly agreed that meetings were productive, their input was valued, their time and expertise were used effectively, and their contributions influenced the direction of the project. Three CAB members found learning about the perspectives of other roles was educational and enlightening. Engaging a CAB with varied roles guided us to co-develop Better Connections, emphasizing the importance of FCG involvement and communication throughout the hospital-SNF transition process.

